# Clinical effect of modified anterolateral acromioarthroplasty during arthroscopic rotator cuff repair

**DOI:** 10.1186/s12891-024-07619-3

**Published:** 2024-07-01

**Authors:** Yongwei Zhou, Jiayu Kang, Qining Yang

**Affiliations:** The Orthopedics Department, Affiliated Jinhua Hospital, Zhejiang University School of Medicine, No. 365 Renmin East Road, Jinhua City, Zhejiang Province 321000 China

**Keywords:** Rotator cuff injury, Anterolateral formation of acromion, Improvement, Rotator cuff retear, Critical shoulder angle

## Abstract

**Background:**

This study aimed to compare the clinical effect of modified anterolateral and traditional acromioplasty in arthroscopic rotator cuff repair.

**Methods:**

The clinical data of 92 patients with total rotator cuff tears admitted to the Department of Joint Surgery of Jinhua Central Hospital from January 2016 to December 2019 were retrospectively analyzed. Among them, 42 patients underwent traditional acromioplasty during arthroscopic rotator cuff repair, and 50 underwent modified anterolateral acromioplasty. Patients were evaluated for preoperative and postoperative shoulder function, pain and critical shoulder angle, and incidence of rotator cuff re-tear at 12 months postoperatively.

**Results:**

The preoperative general data of patients in the classic and modified anterolateral acromioplasty groups did not differ significantly (*P* > 0.05) and were comparable. The UCLA, ASES, and Constant shoulder joint scores were significantly improved in both groups. The VAS score was significantly decreased at 12 months postoperative than preoperative, with a statistically significant difference (*P* ≤ 0.05). Shoulder function and pain scores did not differ significantly between the two groups at 12 months postoperatively (*P* > 0.05). The CSA did not differ significantly between preoperative and postoperative 12 months in the traditional acromioplasty group (*P* > 0.05). However, 12 months postoperative CSA in the modified anterolateral acromioplasty group was significantly smaller than the preoperative CSA, with a statistically significant difference (*P* ≤ 0.05). The rates of rotator cuff re-tears were 16.67% (7/42) and 4% (2/50) in the two groups at 12 months postoperatively, respectively, with statistically significant differences (*P* ≤ 0.05).

**Conclusions:**

Traditional and modified anterolateral acromioplasty while treating total rotator cuff tears using arthroscopic rotator cuff repair significantly improves shoulder joint function. However, modified anterolateral acromioplasty significantly reduced the CSA value and decreased the incidence of rotator cuff re-tears.

## Background

Shoulder joint pain is a common musculoskeletal dysfunction, with an incidence second only to lower back pain [1]. Rotator cuff injury is the leading cause of shoulder joint pain [2, 3]. Neer proposed that 95% of rotator cuff injuries stem from mechanical collisions between the rotator cuff and acromion [4]. Ellman pioneered arthroscopic acromioplasty of the shoulder joint in 1987 and achieved favorable outcomes [5]. Arthroscopic acromioplasty offers advantages, such as shorter surgery duration, smaller surgical scars, better protection of the deltoid muscle, and faster recovery [6]. Consequently, over the past 30 years, acromioplasty during arthroscopic rotator cuff repair has become routine [7]. Many sports medicine practitioners believe that combining effective anterior acromioplasty with rotator cuff repair can safeguard repaired tendons, prevent mechanical wear from postoperative collisions, and reduce the likelihood of re-tearing. However, as research into rotator cuff injuries has advanced, several researchers have questioned the role of traditional anterior lateral acromioplasty. They suggest that performing isolated anterior lateral acromioplasty may cause iatrogenic damage to the coracoacromial arch and deltoid insertion point [8, 9]. With the evolution of arthroscopic techniques, more physicians opt for anterior lateral acromioplasty, which better preserves the coracoacromial arch and deltoid insertion point [10–12]. To our knowledge, few studies have investigated the simultaneous performance of anterior lateral acromioplasty and lateral acromial edge resection in arthroscopic rotator cuff repair. Therefore, this study modified traditional acromioplasty methods to compare the clinical efficacy of these two techniques, providing a more optimized choice for treating rotator cuff injuries.

## Methods

### Inclusion and exclusion criteria

The inclusion criteria were as follows: (1) age ≥ 40 years; (2) patients with unilateral degenerative full-thickness rotator cuff tears diagnosed by X-ray, magnetic resonance imaging (MRI) and shoulder arthroscopy; (3) patients who had undergone standard conservative treatment for three months or more without improvement; (4) preoperative measurement of critical shoulder angle (CSA) > 35° on standard shoulder joint X-ray; (5) patients in generally good condition and capable of tolerating surgery.

The exclusion criteria included the following: (1) patients with rotator cuff injuries due to conditions such as osteoarthritis or trauma; (2) patients with frozen shoulder or shoulder joint infection; (3) patients with massive rotator cuff injuries that are not amenable to repair; (4) patients with a history of previous shoulder surgeries; (5) patients lost to follow-up.

### General information

This study was a retrospective analysis based on existing clinical data. A total of 90 patients were included, all of whom underwent preoperative X-ray and MRI examinations to diagnose rotator cuff injuries. Among them, 42 patients underwent traditional acromioplasty during arthroscopic rotator cuff repair, and 50 underwent modified anterior lateral acromioplasty. This study was approved by the Review Board of Jinhua Central Hospital. Patient-informed consent and ethical review were obtained because this study involved a retrospective analysis of existing imaging data.

**Fig. 1:**
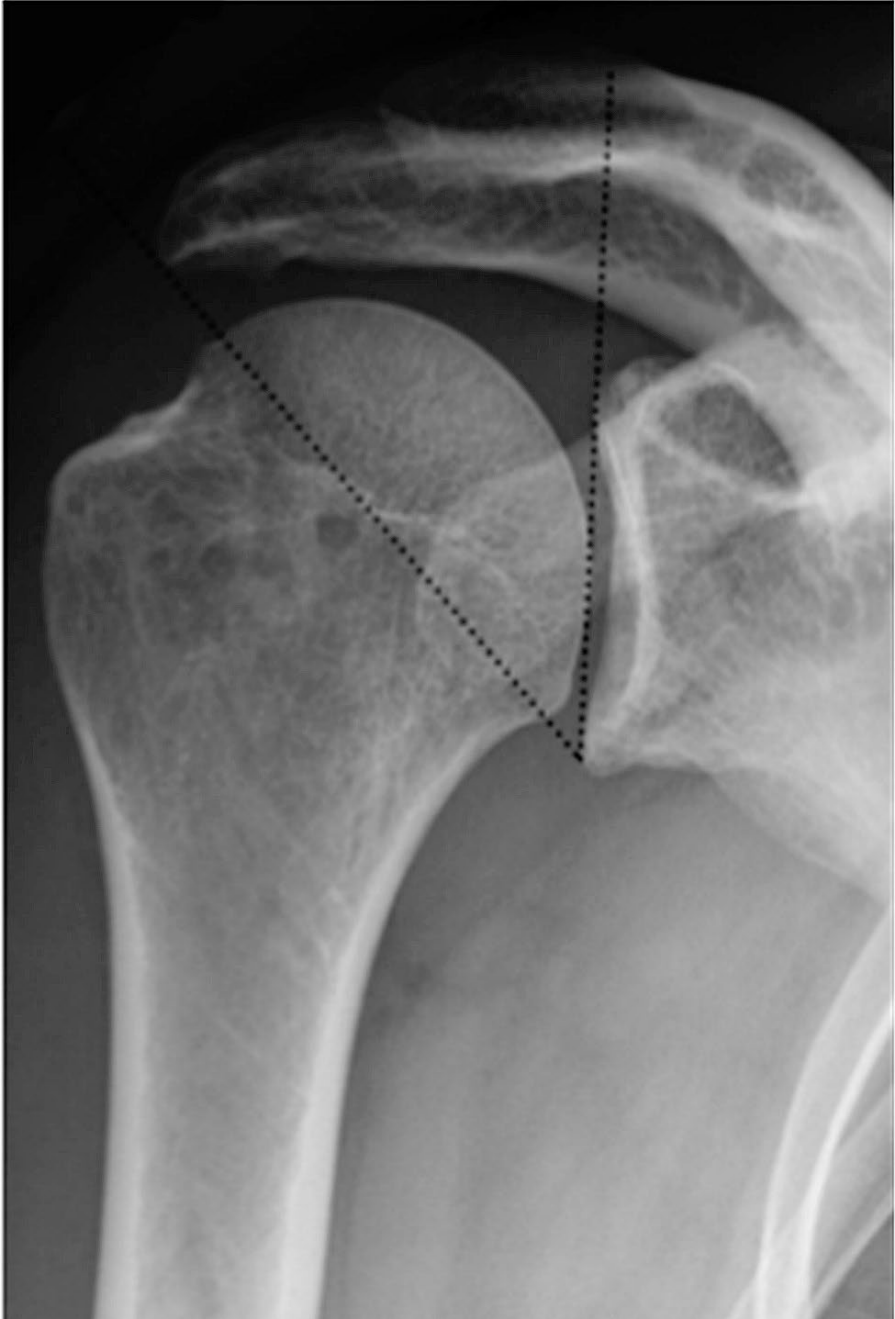
On the anteroposterior X-ray of the shoulder joint, a line is drawn from the upper edge of the pelvis to the lower edge (across the pelvis plane), and a line is drawn from the lower edge of the pelvis to the farthest outer end of the acromion

Comprehensive medical history collection and physical examination were conducted for all patients upon admission. Basic patient information, including age, gender, side of rotator cuff injury, smoking history, diabetes history, and insurance status, was recorded. Preoperative assessments of shoulder joint function (University of California, Los Angeles score-UCLA, American Shoulder and Elbow Surgeons score-ASES, Constant score) and shoulder pain (Visual Analog Scale-VAS) were conducted. The preoperative CSA was measured using standard shoulder X-rays (Fig. 1).

### Surgical procedure

**Fig. 2:**
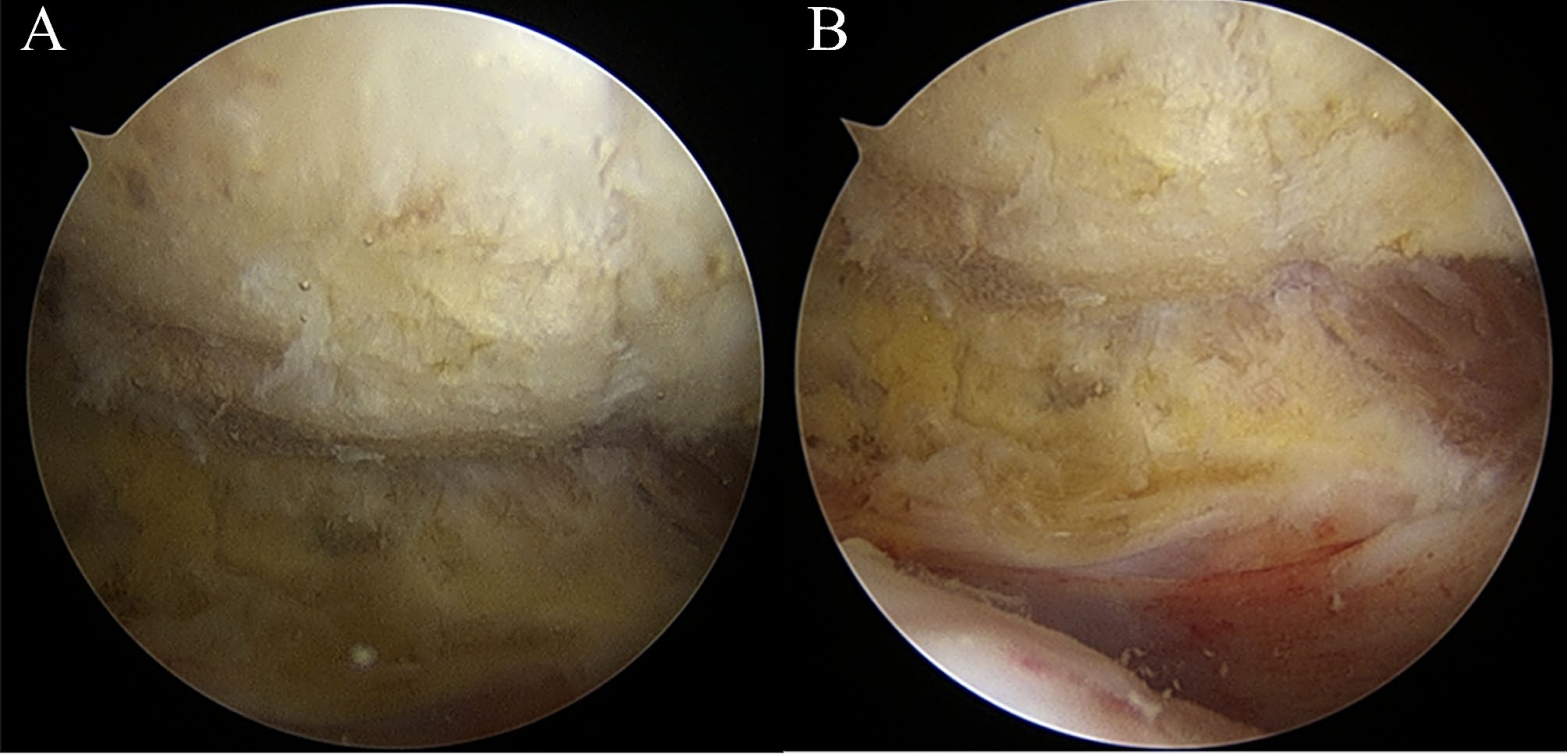
(**A**) Modified Anterior Lateral Acromioplasty. (**B**) Traditional Acromioplasty

Traditional Acromioplasty Group: All patients were placed in a beach chair position under general anesthesia with controlled hypotension. The procedure was performed by the same chief physician who had extensive experience in arthroscopic shoulder surgeries. Standard arthroscopic portals were used to access the joint space via a posterior approach to visually confirm the rotator cuff injuries. Diagnostic examinations of the subacromial space and the glenohumeral joint were conducted via the anterior lateral and anterior portals. An auxiliary portal was established for lateral access under the guidance of the posterior portal, allowing the exposure and cleaning of the subacromial space. A cutting block was employed for traditional anterior acromioplasty. The arthroscope was inserted through the auxiliary portal for observation, and a burr was introduced from the posterior portal to trim and smooth the anterior undersurface of the acromion, removing approximately 5–10 mm of the thickness. Then, the arthroscope was repositioned for further observation to ensure smoothness of the anterior and lateral edges of the acromion (Fig. 2A). Additional trimming and polishing of the anterior undersurface of the acromion were performed. The supraspinatus tendon was repaired using the same suturing technique. Suture incisions were utilized.

Modified Anterior Lateral Acromioplasty Group: Similar to the traditional acromioplasty group, all patients were positioned in a beach chair and received general anesthesia with controlled hypotension. The surgical procedure was performed by the same experienced chief physician. Standard arthroscopic portals were used for joint access via a posterior approach to diagnose rotator cuff injuries. Diagnostic examinations were performed using anterior lateral and anterior portals. The undersurface of the acromion was fully exposed to the lateral edge under the supervision of the posterior portal. Initially, traditional anterior acromioplasty was conducted, followed by using a burr to gradually remove the lateral edge of the acromion from the lateral aspect, ensuring clear visualization of the white tendon endpoint of the supraspinatus muscle in the subacromial space (Fig. 2B). The width of the removed lateral edge was controlled between 5 and 10 mm. After lateral acromioplasty, the footprint of the supraspinatus tendon was exposed and freshened. The torn supraspinatus tendon was repaired using a suture-bridge technique. Suture incisions were employed.

### Postoperative management and efficacy assessment

A standardized rehabilitation protocol was followed for all patients. After surgery, the patients wore an abduction pillow brace for external fixation for four to six weeks. Passive exercises, such as forward flexion and abduction, were performed under the guidance of a physician during this period. Active exercises to protect the shoulder joint were gradually intensified six weeks postoperatively. At 12 weeks postoperatively, active shoulder joint movements were unrestricted.

**Fig. 3:**
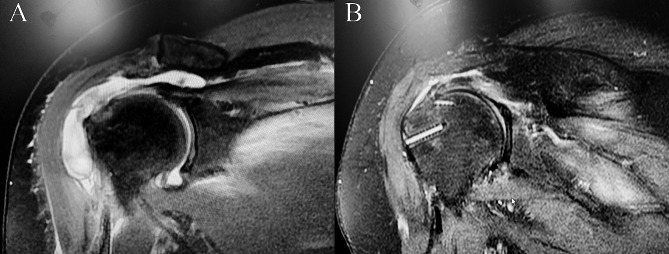
(**A**) Preoperative MR in modified anterolateral plasty group (supraspinatus tendon tear retraction). (**B**) MR at 12 months after operation in modified anterolateral plasty group (Superpost tendon healing)

All patients were scheduled for regular outpatient follow-up appointments postoperatively. At the 12-month follow-up mark, routine shoulder joint X-rays and MRI (Fig. 3) were conducted to assess changes in shoulder joint CSA and the occurrence of rotator cuff re-tear. Functional evaluation of the shoulder joint (UCLA, ASES, and Constant scores) and assessment of shoulder pain (VAS score) were performed at the 12-month follow-up.

### Statistical analysis

IBM SPSS software (version 21.0) was used to process data. The Shapiro-Wilk test was applied to determine whether continuous variables followed a normal distribution. Data were presented as mean ± standard deviation (x ± s). Paired-sample t-tests were used for within-group continuous variables, while independent-sample t-tests were used for between-group continuous variables. The Chi-square test was employed for categorical data. *P*-values ≤ 0.05 were considered statistically significant.

## Results

The baseline characteristics did not differ significantly between the two groups (*P* > 0.05, Table 1), indicating comparability. Shoulder joint UCLA, ASES, VAS, and Constant scores did not differ significantly between the two groups before surgery and 12 months postoperatively (*P* > 0.05, Table 2). However, both groups showed statistically significant increases in shoulder joint UCLA, ASES, VAS, and Constant scores at 12 months postoperatively compared to preoperative values (*P* ≤ 0.05, Table 2). Both groups had significantly lower VAS scores at 12 months postoperatively than preoperative values, with statistically significant differences (*P* ≤ 0.05, Table 2).

**Table 1:** Preoperative general information of patients

	Traditional group(*n* = 42)		Modified group(*n* = 50)	*P*
Gender(%)				0.678
Male	18(42.9%)		24(48.0%)	
Female	24(57.1%)		26(52.0%)	
Age (year), mean ± SD	56.5 ± 15.2		57.6 ± 11.4	0.238
Somke (%)	10(23.8%)		14(28.0%)	0.812
Diabetes (%)	3(7.1%)		2(4.0%)	0.657
Surgical Site (%)				0.668
Left	15(35.7%)		21(42.0%)	
Right	27(64.3%)		29(58.0%)	

*P ≤ 0.05

**Table 2:** UCLA score, ASES score, VAS score, and constant score before and 12 months after surgery in the two groups

	Traditional group(*n* = 42)	Modified group(*n* = 50)	*P*
UCLA score			
Preoperative	12.6 ± 0.8	14.2 ± 1.3	0.425
12 months postoperatively	31.4 ± 3.0	32.0 ± 2.5	0.287
*P*	0.005*	0.001*	
ASES score			
Preoperative	6.8 ± 2.3	6.5 ± 2.1	0.375
12 months postoperatively	13.1 ± 0.7	13.3 ± 0.6	0.428
*P*	0.017*	0.009*	
VAS score			
Preoperative	5.4 ± 1.4	5.2 ± 1.0	0.198
12 months postoperatively	1.5 ± 0.8	1.2 ± 1.1	0.225
*P*	0.004*	0.013*	
Constant score			
Preoperative	31.3 ± 4.3	30.9 ± 7.1	0.764
12 months postoperatively	92.1 ± 6.6	94.3 ± 4.6	0.882
*P*	0.002*	0.000*	

*P ≤ 0.05

**Fig. 4:**
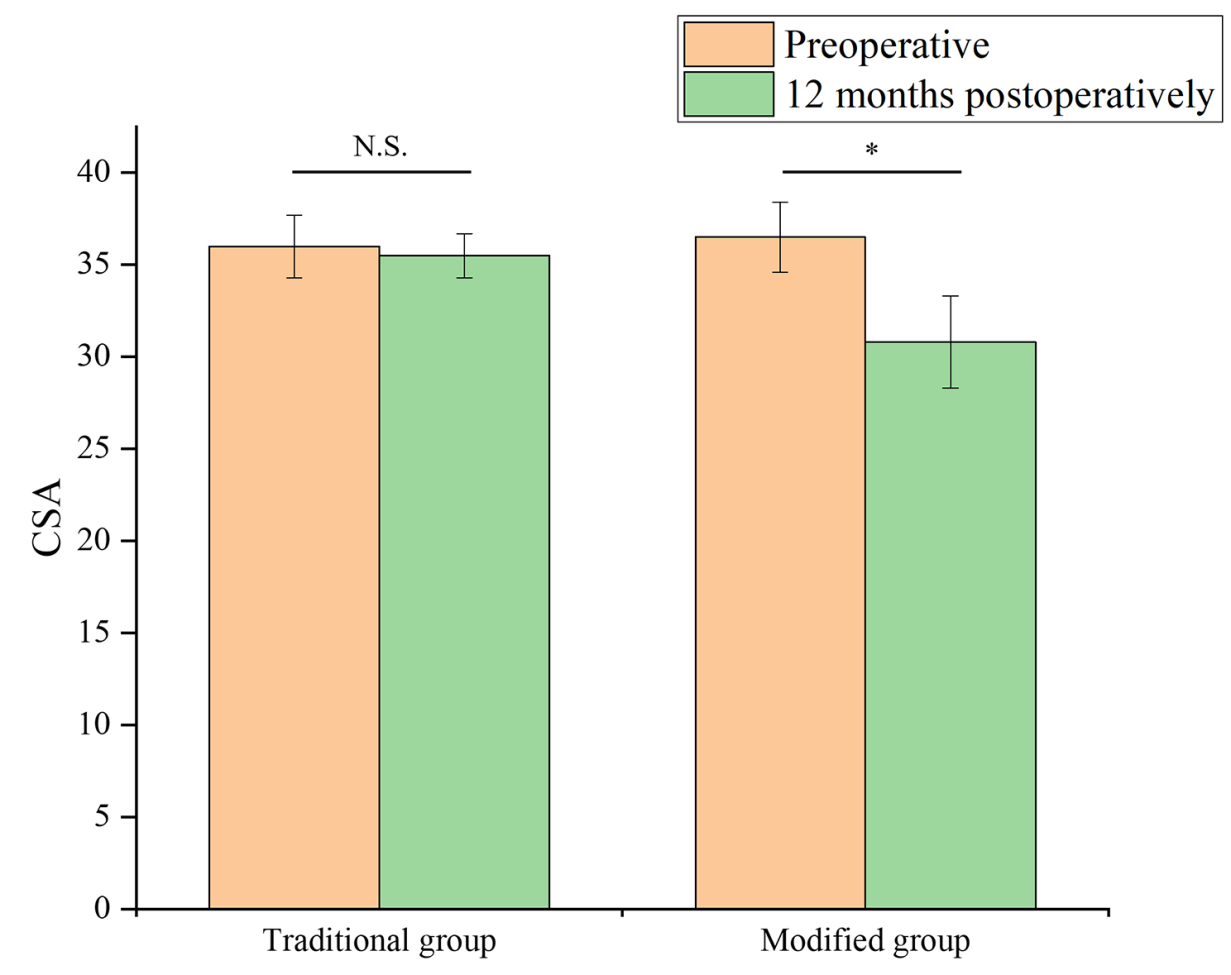
CSA before and 12 months after surgery in Traditional and Modified group. *: *P* < 0.05, N.S.: no significant difference

The traditional acromioplasty group had no significant improvement in CSA at 12 months postoperatively compared to preoperative values(Fig. 4). However, the modified anterior lateral acromioplasty group had significantly reduced CSA 12 months postoperatively compared to the preoperative values, with statistically significant differences (*P* ≤ 0.05, Table 3).

**Table 3:** CSA before and 12 months after surgery, incidence of rotator cuff retear 12 months after surgery in the two groups

	Traditional group(*n* = 42)	Modified group(*n* = 50)	*P*
CSA			
Preoperative	36.0 ± 1.7	36.5 ± 1.9	0.658
12 months postoperatively	35.5 ± 1.2	30.8 ± 2.5	0.023*
*P*	0.327	0.012*	
Retear rate at 12 months after surgery (%)	7(16.67%)	2(4.00%)	0.045*

*P ≤ 0.05

The occurrence rate of rotator cuff re-tear at 12 months postoperatively was 16.67% (7/42) in the traditional acromioplasty group and 4% (2/50) in the modified anterior lateral acromioplasty group, showing a statistically significant difference (*P* ≤ 0.05, Table 3).

## Discussion

### CSA and rotator cuff tears

CSA is a radiological parameter widely used by orthopedic surgeons to analyze pathological changes in the shoulder cuff [13]. In 2013, Moor et al. [4] combined the acromion index and inclination of the glenoid to enhance the sensitivity and accuracy of this parameter for shoulder joint diseases. It is defined as the angle formed between two lines on true anteroposterior X-rays of the shoulder joint. One line connects the upper and lower edges of the glenoid (passing through the glenoid plane), while the other line connects the lower edge of the glenoid to the farthest outer end of the acromion (Fig. 1). The normal range of CSA was 30° to 35°. A value < 30° is associated with shoulder joint osteoarthritis, while a value > 35° is considered a risk factor for full-thickness rotator cuff tears [14]. However, diagnosing rotator cuff tears should not rely solely on a radiological parameter. Song et al. recently conducted a meta-analysis that emphasized the significance of CSA in diagnosing shoulder injuries [15]. Elevated CSA values are related to chronic degenerative full-thickness rotator cuff tears; however, diagnosing these tears is complex and requires comprehensive medical history and clinical physical examinations [16].

### Advantages and necessity of lateral acromial edge resection in rotator cuff repair

Gerber et al. conducted a biomechanical study using a shoulder joint model and found that, as the shoulder joint undergoes abduction, the load on the supraspinatus tendon increases with an increase in CSA [17]. Conversely, reducing the CSA decreases pressure on the supraspinatus tendon. Viehofer et al. suggested a correlation between CSA changes, shoulder joint stability, and shear forces [18]. Increasing the CSA requires greater shoulder cuff activity to maintain joint stability. Therefore, they proposed that reducing excessively high CSA can prevent rotator cuff tears or re-tears. Li et al. indicated that CSA does not affect the postoperative function of arthroscopic rotator cuff repair patients; however, patients with larger postoperative CSA angles face an increased risk of rotator cuff re-tears [19]. Conversely, although anterior acromioplasty can alleviate pain effectively, many studies suggest it cannot prevent rotator cuff tear progression [20]. Some scholars have questioned the necessity of anterior acromioplasty in rotator cuff repair [21]. In our study, performing a modified anterior lateral acromioplasty effectively reduced the length of the lateral acromial edge, thereby lowering the CSA. Therefore, this reduces the pressure load on the supraspinatus tendon during shoulder abduction, effectively slowing down chronic degeneration of the cuff and the occurrence of re-tears. Therefore, this study considered the advantages and necessity of modified anterior lateral acromioplasty. The study results also confirm that cases with lateral acromial edge resection have a lower probability of re-tear, effectively validating this point.

### Precautions for modified anterior lateral acromioplasty

During modified anterior lateral acromioplasty, it is essential to safeguard the integrity of the deltoid insertion and functionality of the deltoid muscle. Gerber et al. conducted a clinical study involving 49 patients who underwent lateral acromial edge resection during rotator cuff repair [22]. The patients were followed up for an average of 30 months. This study found that a resection range of approximately 6 mm at the lateral acromial edge did not significantly affect the deltoid insertion point, as confirmed by preoperative and postoperative MRI comparisons. The impact on deltoid muscle functionality also lacked statistical significance. These findings align with a previous anatomical study by Katthagen et al., demonstrating that a 5 mm resection width at the lateral acromial edge did not affect the deltoid insertion point [23].

Furthermore, subsequent cadaveric studies support this notion. Comparisons with normal specimens have shown that lateral acromial edge resection widths of 5–10 mm do not weaken the function of the acromion or deltoid insertion points [24]. This study controlled lateral acromial edge resection within 10 mm, and postoperative patients did not exhibit significant deltoid atrophy or functional impairments.

4. Indications and Contraindications for Modified Anterior Lateral Acromioplasty.

Indications for modified anterior lateral acromioplasty include repairable full-thickness rotator cuff tears, Type III acromion, and preoperative imaging confirmation of a CSA > 35°. Contraindications mainly included patients with a history of shoulder joint surgery, shoulder joint fractures, dislocations or infections, acromioclavicular arthritis, progressive glenohumeral arthritis, and massive irreparable rotator cuff tears and CSA < 30°.

### limitations of this study

This study has several limitations, as follows. (1) The study was a retrospective study and not a prospective cohort study. It is low quality of evidence provided by a retrospective study. (2) The study focused on full-thickness rotator cuff tears and did not investigate patients with impingement syndrome, partial cuff tears, or massive irreparable cuff tears. (3) The sample size was relatively small. We only included 90 patients in our study, and this could lead to statistical error. (4) Measurement of CSA may have some deviation due to variations in X-ray angles. (5) The follow-up duration after surgery was relatively short, necessitating further follow-up to confirm long-term re-tear rates and functional outcomes. For the limitation, we believe that studies with higher quality of evidence and involving multivariate analysis are required. We hope that our study can provide a basis for future studies with larger sample size, longer-term follow-up outcomes and involving more factors.

## Conclusions

In summary, traditional and modified anterolateral acromioplasty performed while treating total rotator cuff tears using arthroscopic rotator cuff repair significantly improved shoulder joint function. However, modified anterolateral acromioplasty significantly reduced the CSA value and decreased the incidence of rotator cuff re-tears.

## Data Availability

The datasets used and/or analyzed during the current study are available from the corresponding author on reasonable request.

## Abbreviations

CSA: Critical Shoulder Angle
UCLA: University of California, Los Angeles score
ASES: American Shoulder and Elbow Surgeons score
